# Caucasian and Asian Specific Rheumatoid Arthritis Risk Loci Reveal Limited Replication and Apparent Allelic Heterogeneity in North Indians

**DOI:** 10.1371/journal.pone.0031584

**Published:** 2012-02-15

**Authors:** Pushplata Prasad, Ashok Kumar, Rajiva Gupta, Ramesh C. Juyal, Thelma B. K.

**Affiliations:** 1 Department of Genetics, University of Delhi, South Campus, New Delhi, India; 2 Ex-All India Institute of Medical Sciences, New Delhi, India; 3 Department of Rheumatology, Fortis Flt. Lt. Rajan Dhall Hospital, New Delhi, India; 4 Division of Rheumatology & Clinical Immunology, Medanta Bone & Joint Institute, Medanta - The Medicity, Gurgaon, India; 5 National Institute of Immunology, New Delhi, India; South Texas Veterans Health Care System and University Health Science Center San Antonio, United States of America

## Abstract

Genome-wide association studies and meta-analysis indicate that several genes/loci are consistently associated with rheumatoid arthritis (RA) in European and Asian populations. To evaluate the transferability status of these findings to an ethnically diverse north Indian population, we performed a replication analysis. We investigated the association of 47 single-nucleotide polymorphisms (SNPs) at 43 of these genes/loci with RA in a north Indian cohort comprising 983 RA cases and 1007 age and gender matched controls. Genotyping was done using Infinium human 660w-quad. Association analysis by chi-square test implemented in plink was carried out in two steps. Firstly, association of the index or surrogate SNP (r2>0.8, calculated from reference GIH Hap-Map population) was tested. In the second step, evidence for allelic/locus heterogeneity at aforementioned genes/loci was assessed for by testing additional flanking SNPs in linkage equilibrium with index/surrogate marker.

Of the 44 European specific index SNPs, neither index nor surrogate SNPs were present for nine SNPs in the genotyping array. Of the remaining 35, associations were replicated at seven genes namely *PTPN22* (rs1217407, p = 3×10^−3^); *IL2–21* (rs13119723, p = 0.008); *HLA-DRB1* (rs660895, p = 2.56×10^−5^; rs6457617, p = 1.6×10^−09^; rs13192471, p = 6.7×10^−16^); *TNFA1P3* (rs9321637, p = 0.03); *CCL21* (rs13293020, p = 0.01); *IL2RA* (rs2104286, p = 1.9×10^−4^) and *ZEB1* (rs2793108, p = 0.006). Of the three Asian specific loci tested, rs2977227 in *PADI4* showed modest association (p<0.02). Further, of the 140 SNPs (in LE with index/surrogate variant) tested, association was observed at 11 additional genes: *PTPRC, AFF3, CD28, CTLA4, PXK, ANKRD55, TAGAP, CCR6, BLK, CD40* and *IL2RB.* This study indicates limited replication of European and Asian index SNPs and apparent allelic heterogeneity in RA etiology among north Indians warranting independent GWAS in this population. However, replicated associations of *HLA-DRB1, PTPN22* (which confer ∼50% of the heritable risk to RA) and *IL2RA* suggest that cross-ethnicity fine mapping of such loci is apposite for identification of causal variants.

## Introduction

Genome wide association studies (GWAS) have enumerated several new genes/loci for common-complex diseases. Better insight into disease etiology arising from gene discovery via this approach has further fuelled the hope that risk models based on these findings will lead to personalized and preventive medicine and also therapeutic interventions of complex diseases. Replications of GWAS findings across ethnic groups albeit with varying effect sizes fortify this expectation.

Recent GWAS in rheumatoid arthritis (RA; MIM180300) have unraveled disease susceptibility loci of small to moderate effect size. Most of these genes/loci have risk alleles of known immune function [1] justifying their involvement in RA which is a complex autoimmune disorder characterized by chronic inflammation of the synovial joints followed by progressive articular damage and major functional disability [2]. Approximately 1% of the adult population worldwide is affected by RA. However, the prevalence varies from 0.2–0.3% in population from south-east Asia to 6% in native American-Indian populations (i.e., Pima and Chippewa Indians [3]) and women twice as likely to develop the disease as men [2]. Findings from studies carried out in two Northern European regions have suggested that approximately 60% of the disease variance can be attributed to heritable factors [4]. Genetic association studies have long implicated the human leukocyte antigen locus DRB1 as the principle genetic factor conferring risk to RA. In addition to HLA, GWAS carried out in European populations have identified a total of 24 susceptibility genes/loci (having 26 risk alleles) almost consistently associated with RA. These include *PTPN22, 6q23, TRAF1/C5, STAT4, IL2RB, KIF5A, PRKCQ, IL2_IL21, CD226, CCL21, CD40, CTLA4, IL2RA, AFF3, IL7R*, *BLK* and *c-Rel*. Further, a recent GWAS using a meta-analysis approach re-confirmed the importance of these 26 RA risk alleles (Table S1) among Europeans [5]. The study also identified seven new risk alleles conferring susceptibility to RA in European ancestry. These SNPs are present in close proximity to genes of known immune function, namely, *IL6ST, SPRED2, RBPJ, CCR6, IRF5* and *PXK*. However, *HLA-DRB1* and *PTPN22* together explain around 50% of the heritable risk to RA [6] and the effects of other genes are weak (odds ratio (OR) <1.3). It is noteworthy that while alleles at *HLA-DRB1* locus, the largest predisposing genetic risk factor to RA, has been associated with RA among both Caucasian and Asian populations, not all European specific RA susceptibility risk loci are associated among Asians. The risk allele of SNP R620W in *PTPN22* is monomorphic in Asian ethnicity [7]–[8]. On the other hand, SNPs at Asian specific RA loci i.e., *PADI4, SLC22A4, and FCRL3* [9] showed either modest or no association in the meta-analysis [5] and other association studies reported in European populations [10]–[12]. These findings point towards genetic heterogeneity in RA susceptibility across different ethnic groups.

RA is observed with same prevalence in the genetically distinct north Indian population. Contribution of HLA locus to disease in the population is well documented [13]–[14]. However, concrete data on contribution of other candidate genes/loci are lacking. Therefore, we investigated association status of RA risk conferring genes/loci identified in European meta-analysis [5] in the ethnically distinct north-Indian cohort using a two pronged approach. Firstly, we carried out association analysis of 44 SNPs from 40 candidate genes/loci (33 risk alleles with genome wide significance i.e., p<10-8 and 11 moderate associations reported in the European meta-analysis [5] and also three Asian specific genes (*PADI4, SLC22A4, and FCRL3*) mentioned above. In the second step, evidence for allelic/locus heterogeneity at aforementioned genes/loci was assessed for by testing additional flanking SNPs in linkage equilibrium with index/surrogate marker. The study reports limited replication of European and Asian specific index SNPs and apparent allelic heterogeneity in RA etiology among north Indians.

## Results

### i) Replication of association of 47 index/surrogate SNPs from 43 candidate genes/loci

#### a) SNPs reported in European population

Only 21 out of the 44 European specific index SNPs (from 40 genes/loci) were present in the SNP array used in this study. Of the remaining 23 SNPs, surrogates were identified for 14 index variants but none for nine SNPs (rs10865035, rs10499194, rs5029937, rs3218253, rs934734, rs6859219, rs13315591, rs874040, rs840016, and rs7155603) [Table S1]. Despite the presence of index SNP rs2476601 in *PTPN22*, but with its largely monomorphic status in our cohort, a surrogate SNP rs1217407 was identified (and tested for association). Associations (p<0.05) were replicated at only seven loci namely *PTPN22-*rs1217407, *IL2–21-*rs13119723, *HLA-DRB1-*rs6910071, *TNFA1P3-*rs9321637, *CCL21-*rs13293020, *IL2RA-*rs2104286 and *ZEB1-*rs2793108. Although, the European meta-analysis index SNP (rs6910071) from *HLA-DRB1* showed marginal association (p = 0.046) in our cohort, further testing of other SNPs from this gene (Table S1) revealed significant association of three SNPs with RA in our population.

#### b) SNPs reported in Asian population

rs3761959 and rs3753389 from *FCRL3,* and *CD244* respectively were not associated with RA in our cohort. Index SNP rs11203367 in *PADI4* was not present on the SNP array used in the study but the surrogate SNP (rs2977227) showed association (p<0.02) with RA (Table S1).

### ii) Evaluation of allelic/genetic locus heterogeneity at 42 (39 European and 3 Asian) candidate genes/loci

#### a) 39 candidate genes/loci from European meta-analysis

To evaluate allelic heterogeneity in the potential candidate genes/loci, a total of 603 SNPs flanking index/surrogate SNPs (selected based on LE) spanning across the 39 genes/loci (excluding HLA) were tested (Table S1 and Table S2). Besides significant associations at seven markers mentioned above, associations (p<0.05) were observed with SNPs in 11 additional genes namely, rs9803750 and rs2359952 (p<5×10-4) in *PTPRC*; rs17023158, rs6706188, and rs1437377 (p<5×10-3) in *AFF3*; rs4675367 (p<0.05) in *CD28*; rs231726 and rs10197319 (p = 0.03) in *CTLA4*; rs7622074 (p<0.02) and rs6767498 (p<0.005) in *AFF3*; rs6877664 (p = 0.016), rs10214316 (p = 0.005), rs149140 (p = 0.008) and rs32498 (p = 0.006) in *ANKRD55*; rs926657 (p = 0.003), rs9295089 (p = 0.009), rs212402 (p = 0.009) in *TAGAP*; rs1331301 (p = 0.01) and rs1556413 (p = 0.02) in *CCR6*; rs4841548 (p = 0.03) and rs17806523 (p = 0.009) in *BLK*; rs6065925 (p<0.005) in *CD40*; and rs228942 (p<0.05) in *IL2RB* (Table S1). In addition, two SNPs (rs3757173 and rs5029936) in *TNFA1P3* also showed stronger association (p<0.03) than the surrogate SNP (p<0.05) (Table S1).

#### b) 3 candidate genes from Asian population

No association of any SNP from the three Asian specific genes was observed.

**Figure 1:**
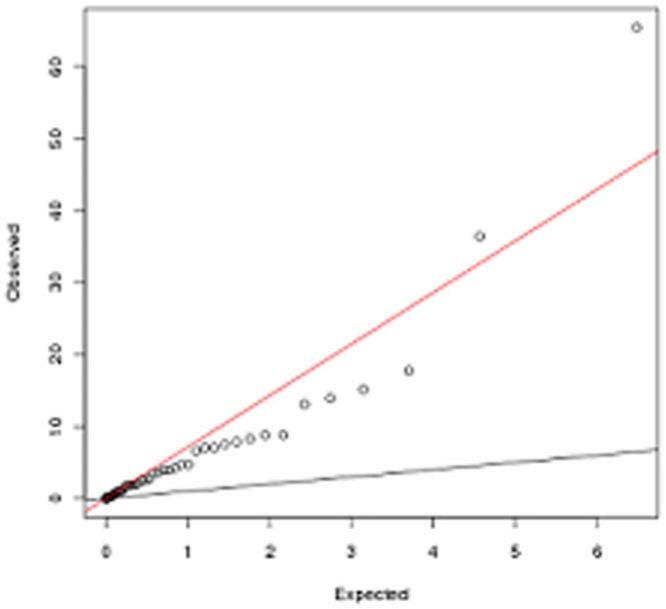
Quantile-Quantile plot of chi-square test of association p-values of 48 SNPs (presented in Table S1).

Thus of the 40 genes/loci showing disease association among Europeans and of the three among Asians, replication was observed at only 18 and one gene (*PADI4*) respectively in the north Indian cohort. Further, of the above mentioned 19 associated markers only four SNPs withstood Bonferroni's correction (Table S1, Figure 1).

With 983 cases/1007 controls and after accounting for 173 comparisons (alpha set at 0.0003), the study had sufficient power (80%) to detect associations with odds ratios 1.3 or higher (or 0.77 or lower) for allele frequencies between 20%∼30%, odds ratios 1.4 or higher (0.71 or lower) for allele frequencies between 10%–20% & odds ratios 1.6 or higher for allele frequencies of 5∼10% assuming a log-additive model of inheritance.

## Discussion

Recent GWAS have identified 40 susceptibility genes/loci in European and three in Asian populations that confer predisposition to RA (Table S1). Reported genetic associations but with varying effect sizes and replicated across populations may reiterate the importance of these genes in disease etiology independent of the environmental attributes. Conversely, non-replication would be an impetus for novel gene(s) discovery. Limited replication of the CEU meta-analysis findings in RA among the ethnically distinct north-Indian cohort in this study is noteworthy.

Replication of association of SNPs in *HLA-DRB1, IL2–21 and IL2RA* (Table S1) clearly testify that the immune genes are major and common players in RA across diverse ethnicities. HLA*-DRB1* shared epitope (SE) acts as an immune-stimulatory ligand that can direct T cell differentiation toward Th17 cells (that are) implicated in the pathogenesis of autoimmune diseases, including RA [15]. Marginal association of the index SNP and strong association of three other SNPs which have been shown to be strongly associated with the disease in other Caucasian and Korean studies [6], [16]–[17] verify the importance of HLA-DRB1 in our cohort. *IL2–21* and *IL2RA* are cytokines with immuno-regulatory activity and are considered general susceptibility loci for inflammatory diseases. Another gene *ZEB1* (found moderately associated (p = 2×10-3) in European meta-analysis), believed to play a role in transcriptional repression of *IL2* pathway also showed significant association (p = 6×10-3) in our study. Absence/marginal association of index/surrogate SNPs but stronger association of flanking SNPs at RA risk loci in our study cohort (Table S1) is suggestive of allelic heterogeneity and warrants discussion.

Among the non-HLA markers, the mis-sense 620Arg>Trp (rs2476601) variant in *PTPN22* is the most strongly associated SNP in various RA association studies performed in Caucasian populations. The disease-associated allele (Trp) prevents the interaction of the lymphoid protein tyrosine phosphatase (LYP) with the T cell receptor-associated kinases. This may increase the overall reactivity of the immune system thus predisposing an individual to autoimmune disease. This SNP was largely monomorphic (MAF<0.02) in our population in conformity with the other reports from Asia [7]–[8]. However, unlike the results from an extended analysis of the *PTPN22* locus among Koreans [8] which reported absence of association of any other SNP from this gene, a significant association of rs1217407 (p = 3.0×10-3) with RA was observed in our study (Table S1). Though, this may be suggestive of allelic heterogeneity, significance of this intronic SNP remains unexplained to date. Similarly significant association of flanking SNPs were observed in *PTPRC, AFF3, CD28, CTLA4, PXK, ANKRD55, TAGAP, CCR6, BLK, CD40* and *IL2RB* believed to have immuno-regulatory functions. Except for non-synonymous polymorphism rs228942 (Asp391Glu) in *IL2RB*, other SNPs lie either in the intronic or 3′UTR region of the associated gene and thus functional significance remain to be validated. Alternatively, the associated SNP(s) may be in LD with another causal variant(s) yet unidentified. These findings together suggest the role of all the genes in RA among north-Indians but with likely heterogeneity and varied effect sizes.

**Figure 2:**
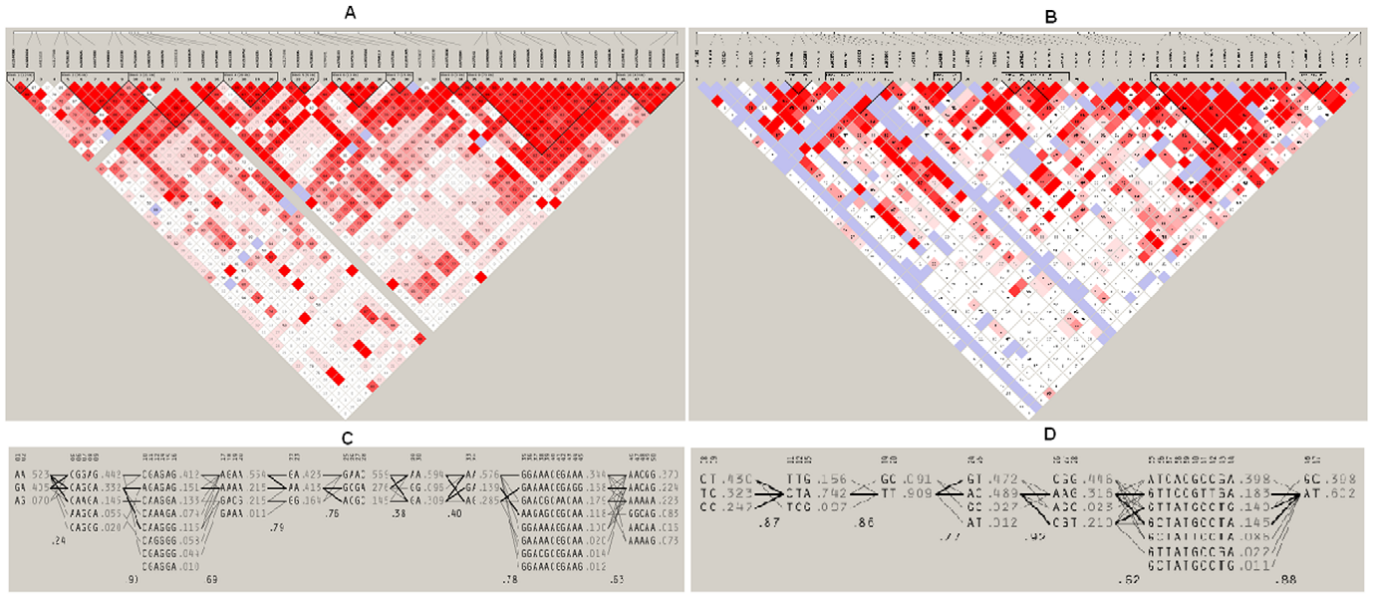
A) LD plot of *PTPRC* among north-Indians, B) LD plot of *PTPRC* among CEU population, C) *PTPRC* haplotypes among north-Indians, D) *PTPRC* haplotypes among CEU population.

**Figure 3:**
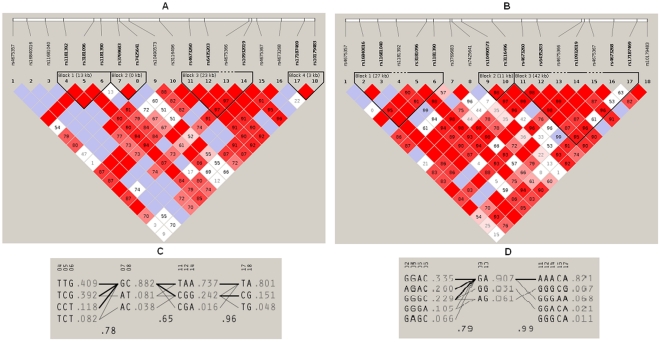
A) LD plot of *CD28* among north-Indians, B) LD plot of *CD28* among CEU population, C) *CD28* haplotypes among north-Indians, D) *CD28* haplotypes among CEU population.

Failure to replicate association with the majority of genes/loci reported from European ancestry is unlikely the result of Type II error, given the sample size (n∼1000 cases and 1000 controls each) and more than 80% power therein to detect association at moderate effect size. Second reason for non-replication could be that the risk alleles reported in European populations may play a role in susceptibility to RA only in the presence of certain population specific and as yet unidentified environmental triggers/diet/specific infectious agents. Alternatively, the index SNPs may not be the causal SNPs, but could be in linkage disequilibrium with the true disease causing SNPs on a haplotype that is found in the European population but not in the north-Indian cohort. On the other hand, our results may reflect true genetic heterogeneity underlying RA. This hypothesis draws support from a recently published article on comparative population architecture among Indians and other HapMap populations [18]. A comparison of 55 diverse endogamous populations from India with CEU, YRI, CHB, JPT populations (HapMap database) reflected the dissimilarity/genetic heterogeneity among Caucasian (CEU), Asians (CHB/JPT), and north-Indian populations. The principal component analysis suggested that although the north-Indian population shows maximum proximity to CEU as compared to all other HapMap populations, they still form separate clusters. As for population stratification in the study sample which may influence genetic association results, every precaution was taken to carefully match the controls and the patients based on age, sex, and self reported ethnicity over the last three generations. Further, less suitability of array design (poor tag transferability) due to population specific LD patterns across susceptibility loci may explain limited capture of causal variants in the north Indian cohort. This possibility draws support from findings previously reported from the lab for ulcerative colitis [19] and is reiterated in this study. To illustrate this, a comparative LD profiles for representative genes namely *PTPRC* and *CD28* are shown in Figures 2 & 3. Smaller LD blocks in our north-Indian (NI) cohort as compared to CEU population are obvious in these LD plots.

In conclusion, our findings of poor/non-replication of European GWAS hits in the north Indian cohort suggest differences in genetic architecture between populations; they also reiterate that in addition to common genetic risk factors, such as the HLA*– DRB1*, there exist population specific genes involved in the etiology of RA, necessitating GWAS in multiple ethnic groups. This is supported by another recent study from our group reporting limited replication of European findings in an Ulcerative colitis cohort from north India [19]. These results emphasise the need for finer/additional genome analysis of less investigated ethnic groups which may greatly facilitate identification of novel genes besides precise identification of disease causing variants in known genes.

## Materials and Methods

### Ethics Statement

The study was approved by the ethical committees of the participating institutions (University of Delhi South Campus, New Delhi; All India Institute of Medical Sciences, New Delhi; Dayanand Medical College & Hospital, Ludhiana). Informed written consent was obtained from all the subjects who participated in this study. The study follows the recommendations of the Declaration of Helsinki (2008).

### RA and control subjects

Demographic Details: Ethnicity/Origin of individuals included in the study cohort was ascertained by their language (mother tongue) and geographical zone. The study cohort comprised of subjects who are north-Indian (from the northern states of India namely Jammu and Kashmir, Punjab, Haryana, Himachal Pradesh, Uttaranchal, Uttar Pradesh, Bihar, Jharkhand, and Madhya Pradesh) by ethnicity at least over the last three generations.

A total of n = 983 RA cases of north-Indian origin were recruited from the All India Institute of Medical Sciences (AIIMS) and Research and Referral Hospital (R&R), New Delhi based on American College of Rheumatology criteria for RA [20]. Sera were stored at −70°C and tested for sero-subtype of rheumatoid factor (IgG, IgM and IgA) and anti-CCP antibodies (using standard protocols) in one sitting after the recruitment of patients was complete. C reactive protein (CRP), and erythrocyte sedimentation rate (ESR) were also measured. N = 1007 healthy controls which included spouses and healthy staff members (above 35 years of age with no medical history of RA/or any other form of arthritis) of north-Indian origin were recruited from the study hospitals mentioned above and also from Dayanand Medical College & Hospital, Ludhiana.

### DNA extraction, Genotyping and Quality Control

DNA was collected from peripheral blood samples of RA patients and control samples using conventional phenol-chloroform method. Genotyping was carried out using Infinium Human 660W-quad SNP microarray platform. The average genotyping success rate was 99.98%. To this data we subsequently applied several quality control filters before SNPs and individuals were included in the final analysis:

1. exclusion of SNPs with more than 5% of the values missing, exclusion of individuals with more than 5% of genotyping values missing (none), exclusion of SNPs with a minor allele frequency (MAF) <0.05, exclusion of SNPs failing Hardy Weinberg Equilibrium test (p<0.0001).
2. Pi-HAT (pair-wise pi-Hat<0.2) test for relatedness was carried out to exclude related individuals.
3. Multi-dimensional scaling (MDS) analysis was carried out to (i) exclude outliers from the study cohort and (ii) to rule out population stratification/genetic heterogeneity.

A total of 47 SNPs were initially selected for analysis in this study. SNPs included were either index SNPs from the European GWAS meta-analysis study [5] or their surrogates in LD (r2>0.8, as seen in GIH Hap-Map Phase III population) when index SNPs were not covered in the array used in the study. To preclude the possibility of allelic/locus heterogeneity for such genes/loci, additional SNPs in linkage equilibrium (LE) with index/surrogate markers (based on GIH Hap-Map Phase III population) in candidate genes/loci (Table S1), were analysed. Basic allelic association test (which compares frequencies of alleles among case and control groups) by chi-square test implemented in PLINK [21] was performed to test association between a SNP and disease (rheumatoid arthritis). To ascertain significance of association odds ratio at 95% confidence interval was also calculated. Quanto software was used to estimate power of the study (University of South California, http://hydra.usc.edu/gxe). Odds ratios (OR) considering a Bonferroni's correction P-value of 0.0003 (for 173 comparisons) and different allele frequencies were calculated.

## Supporting Information

Table S1 **Association analysis of European specific RA susceptibility genes/loci in north Indian cohort.**
(DOC)

Table S2 **Test of association of additional (flanking) SNPs in European specific RA candidate genes/loci.**
(DOC)
