# Supplementary material for: Caucasian and Asian Specific Rheumatoid Arthritis Risk Loci Reveal Limited Replication and Apparent Allelic Heterogeneity in North Indians
Source: PLoS One. 2012 Feb 15;7(2):e31584. doi: 10.1371/journal.pone.0031584 (PMC3280307; doi:10.1371/journal.pone.0031584)
Supplement: Table S1 — Association analysis of European specific RA susceptibility genes/loci in north Indian cohort. (DOC) [file pone.0031584.s001.doc]

| **Locus** | **ID** | **Gene(s)** | ***PGWAS*** | **P (NI)** | **Minor Allele** | **OR** | **L95** | **U95** | **Additional associations** | **P** | **Minor Allele** | **OR** | **L95** | **U95** |
| --- | --- | --- | --- | --- | --- | --- | --- | --- | --- | --- | --- | --- | --- | --- |
| 1p36 | rs3890745 | *TNFRSF14* | 3.6 × 10^−6^ | 0.161 | G | 0.91 | 0.81 | 1.04 |  |  |  |  |  |  |
| 1p13 | rs2476601 | *PTPN22* | 9.1 × 10^−74^ | 0.76 | A | 0.94 | 0.63 | 1.4 |  |  |  |  |  |  |
|  | rs1217407* |  |  | **3.0 X 10-3** | A | 0.8 | 0.69 | 0.93 |  |  |  |  |  |  |
| 1p13 | rs11586238 | *CD2, CD58* | 1.0 × 10^−5^ | NA |  |  |  |  |  |  |  |  |  |  |
|  | rs12405671* | *CD2, CD58* |  | 0.21 | A | 1.1 | 0.95 | 1.28 |  |  |  |  |  |  |
| 1q23 | rs12746613 | *FCGR2A* | 0.0004 | NA |  |  |  |  |  |  |  |  |  |  |
|  | rs12722986* |  |  | 0.84 | G | 1.02 | 0.81 | 1.28 |  |  |  |  |  |  |
| 1q31 | rs10919563 | *PTPRC* | 0.0002 | NA |  |  |  |  | rs9803750 | **4.3 x 10^-4^** | G | 0.79 | 0.69 | 0.90 |
|  | rs1011338* |  |  | 0.60 | G | 0.96 | 0.82 | 1.11 | rs2359952 | **1.6 x 10^-4^** | G | 0.78 | 0.68 | 0.88 |
| 2p14 | rs934734 | SPRED2 | 3.2 × 10^−7^ | NA, NS |  |  |  |  |  |  |  |  |  |  |
| 2p16 | rs13031237 | *REL* | 7.9 × 10^−7^ | 0.42 | A | 0.92 | 0.74 | 1.13 |  |  |  |  |  |  |
| 2q11 | rs10865035 | *AFF3* | 2.0 × 10^−6^ | NA |  |  |  |  | rs17023158 | **0.005** | G | 1.45 | 1.12 | 1.88 |
|  |  |  |  |  |  |  |  |  | rs6706188 | **0.005** | G | 0.81 | 0.7 | 0.94 |
|  |  |  |  |  |  |  |  |  | rs1437377 | **0.003** | C | 0.77 | 0.65 | 0.92 |
| 2q32 | rs7574865 | *STAT4* | 2.9 × 10^−7^ | 0.33 | A | 1.07 | 0.93 | 1.23 |  |  |  |  |  |  |
| 2q33 | rs1980422 | *CD28* | 5.2 × 10^−5^ | NA |  |  |  |  | rs4675367 | **0.041** | A | 1.8 | 1.02 | 3.19 |
|  | rs3116496* | *CD28* |  | 0.63 | G | 1.05 | 0.85 | 1.31 |  |  |  |  |  |  |
| 2q33 | rs3087243 | *CTLA4* | 1.2 × 10^−8^ | NA |  |  |  |  | rs231726 | **0.03** | G | 1.16 | 1.01 | 1.33 |
|  | rs231804* | *CTLA4* |  | 0.18 | A | 1.09 | 0.96 | 1.24 | rs10197319 | **0.03** | A | 1.15 | 1.01 | 1.3 |
| 3p14 | rs13315591 | PXK | 3.7 × 10^−7^ | NA, NS |  |  |  |  | rs7622074 | 0.011 | G | 1.18 | 1.04 | 1.34 |
|  |  |  |  |  |  |  |  |  | rs6767498 | 0.005 | A | 0.83 | 0.73 | 0.95 |
| 4q27 | rs6822844 | *IL2, IL21* | 0.0007 | NA |  |  |  |  |  |  |  |  |  |  |
|  | rs13119723* | *IL2, IL21* |  | **0.008** | G | 0.75 | 0.61 | 0.93 |  |  |  |  |  |  |
| 4p15 | rs874040 | RBPJ | 1.9 × 10^−7^ | NA, NS |  |  |  |  |  |  |  |  |  |  |
| 5q11 | rs6859219 | ANKRD55, IL6ST | 2.5 × 10^−9^ | NA, NS |  |  |  |  | rs6877664 | **0.016** | A | 1.41 | 1.06 | 1.88 |
|  |  |  |  |  |  |  |  |  | rs10214316 | **0.005** | G | 1.2 | 1.06 | 1.37 |
|  |  |  |  |  |  |  |  |  | rs149140 | **0.008** | A | 1.19 | 1.05 | 1.35 |
|  |  |  |  |  |  |  |  |  | rs32498 | **0.006** | G | 1.2 | 1.05 | 1.36 |
| 5q21 | rs26232 | C5orf30 | 4.3 × 10^−7^ | 0.79 | T | 0.98 | 0.83 | 1.15 |  |  |  |  |  |  |
| 6p21 | rs6910071 | *HLA-DRB1 (*0401 tag)* | <10−299 | **0.04** |  | 1.27 | 1 | 1.61 |  |  |  |  |  |  |
| 6p21 | rs660895** | *HLA-DRB1* | 1 x 10-108 | **2.56 X 10-5** | G | 1.52 | 1.25 | 1.85 |  |  |  |  |  |  |
| 6p21 | rs6457617** | *HLA-DRB1* | 1 x 10^-9^ | **1.6 X 10-9** | A | 1.48 | 1.3 | 1.69 |  |  |  |  |  |  |
| 6p21 | rs13192471** | *HLA-DRB1* | 2 x 10^-58^ | **6.7 X 10-16** | G | 2.16 | 1.79 | 2.61 |  |  |  |  |  |  |
| 6q21 | rs548234 | *PRDM1* | 9.7 × 10^−5^ | 0.33 | G | 1.08 | 0.92 | 1.26 |  |  |  |  |  |  |
| 6q23 | rs10499194 | *TNFAIP3* | 0.0007 | NA |  |  |  |  | rs3757173 | **0.03** | G | 1.27 | 1.02 | 1.58 |
| 6q23 | rs6920220 | *TNFAIP3* | 8.9 × 10^−13^ | NA |  |  |  |  | rs5029936 | **0.03** | A | 1.34 | 1.03 | 1.74 |
|  | rs9321637* |  |  | **0.04** | G | 0.76 | 0.59 | 0.97 |  |  |  |  |  |  |
| 6q23 | rs5029937 | *TNFAIP3* | 7.5 × 10^−8^ | NA |  |  |  |  |  |  |  |  |  |  |
| 6q25 | rs394581 | *TAGAP* | 0.0006 | NA |  |  |  |  | rs926657 | **0.003** | A | 0.65 | 0.49 | 0.85 |
|  | rs169858* |  |  | 0.15 | A | 0.89 | 0.76 | 1.04 | rs9295089 | **0.009** | G | 0.71 | 0.55 | 0.92 |
|  |  |  |  |  |  |  |  |  | rs212402 | **0.009** |  | 0.82 | 0.70 | 0.95 |
| 6q27 | rs3093023 | CCR6 | 3.3 × 10^−7^ | 0.07 | A | 1.13 | 0.99 | 1.28 | rs1331301 | 0.01 | C | 0.85 | 0.75 | 0.97 |
|  |  |  |  |  |  |  |  |  | rs1556413 | 0.02 | A | 0.86 | 0.76 | 0.98 |
| 7q32 | rs10488631 | IRF5 | 2.8 × 10^−6^ | 0.79 | C | 0.98 | 0.83 | 1.15 |  |  |  |  |  |  |
| 8p23 | rs2736340 | *BLK* | 1.5 × 10^−5^ | 0.12 | A | 1.11 | 0.97 | 1.27 | rs4841548 | **0.03** | G | 0.83 | 0.69 | 0.98 |
|  |  |  |  |  |  |  |  |  | rs17806523 | **0.009** | A | 0.73 | 0.58 | 0.93 |
| 9p13 | rs2812378 | *CCL21* | 0.0001 | NA |  |  |  |  |  |  |  |  |  |  |
|  | rs13293020* |  |  | **0.01** | A | 0.7 | 0.53 | 0.92 |  |  |  |  |  |  |
| 9q33 | rs3761847 | TRAF1, C5 | 2.1 × 10^−7^ | 0.95 | G | 1 | 0.88 | 1.15 |  |  |  |  |  |  |
| 10p15 | rs2104286 | *IL2RA* | 0.002 | **1.9 × 10^−4^** | G | 0.73 | 0.62 | 0.86 | rs12722589 | **7.4X10-5** | C | 1.48 | 1.22 | 1.80 |
|  |  |  |  |  |  |  |  |  | rs3118470 | **2.0x 10^-3^** | G | 1.22 | 1.07 | 1.38 |
| 10p15 | rs4750316 | *PRKCQ* | 2.0 × 10^−6^ | NA |  |  |  |  |  |  |  |  |  |  |
|  | rs10796045* |  |  | 0.09 | C | 0.88 | 0.75 | 1.02 |  |  |  |  |  |  |
| 11p12 | rs540386 | *TRAF6* | 0.0003 | NA |  |  |  |  |  |  |  |  |  |  |
|  | rs1046864* |  |  | 0.3 | A | 0.91 | 0.77 | 1.08 |  |  |  |  |  |  |
| 12q13 | rs1678542 | *KIF5A, PIP4K2C* | 0.0002 | NA |  |  |  |  |  |  |  |  |  |  |
|  | rs11172254* |  |  | 0.47 | A | 0.95 | 0.84 | 1.08 |  |  |  |  |  |  |
| 20q13 | rs4810485 | *CD40* | 2.8 × 10^−9^ | NA |  |  |  |  | rs6065925 | **0.005** | A | **1.12** | 1.06 | 1.38 |
|  | rs6074022* |  |  | 0.06 | G | 0.87 | 0.75 | 1.01 |  |  |  |  |  |  |
| 22q12 | rs3218253 | *IL2RB* | 0.002 | NA, NS |  |  |  |  | rs228942 | **0.04** | A | 0.84 | 0.7 | 0.99 |
| **Association status of 11 RA susceptibility genes/loci showing moderate association in GWAS Meta-analysis** | | | | | | | | | | | | | | |
| 1q21 | rs7543174 | IL6R | 7.9 × 10^−5^ | 0.6 | C | 1.05 | 0.88 | 1.24 |  |  |  |  |  |  |
| 1q24 | rs840016 | CD247 | 3.6 × 10^−5^ | NA, NS | T |  |  |  |  |  |  |  |  |  |
| 4q27 | rs13119723 | IL2, IL21 | 0.001 | **0.008** | G | 0.75 | 0.61 | 0.93 |  |  |  |  |  |  |
| 10p15 | rs11594656 | IL2RA | 0.0002 | 0.37 | A | 1.08 | 0.91 | 1.27 |  |  |  |  |  |  |
| 10p11 | rs2793108 | ZEB1 | 0.002 | **0.006** | C | 0.84 | 0.74 | 0.95 |  |  |  |  |  |  |
| 12q24 | rs3184504 | SH2B | 0.004 | 0.19 | C | 1.14 | 0.93 | 1.39 |  |  |  |  |  |  |
| 14q24 | rs7155603 | BATF | 1.0 × 10^−5^ | NA, NS | G |  |  |  |  |  |  |  |  |  |
| 16p11 | rs8045689 | CD19, NFATC2IP | 5.3 × 10^−5^ | 0.73 | C | 1.03 | 0.88 | 1.21 |  |  |  |  |  |  |
| 17q12 | rs2872507b | IKZF3 | 4.7 × 10^−5^ | 0.86 | A | 0.99 | 0.87 | 1.13 |  |  |  |  |  |  |
| 21q22 | rs11203203b | UBASH3A | 2.5 × 10^−5^ | 0.17 | A | 1.11 | 0.96 | 1.26 |  |  |  |  |  |  |
| 22q11 | rs5754217b | UBE2L3 | 0.0007 | 0.11 | T | 1.11 | 0.97 | 1.26 |  |  |  |  |  |  |
|  |  |  |  |  |  |  |  |  |  |  |  |  |  |  |

*Surrogate SNPs, **HLA-DRB1 alleles found significant in other GWAS [Reference 6, 17, 18], NA: Index SNP not available on the array used in our study, NS: No Surrogate available for the SNP
