# Supplementary material for: Caucasian and Asian Specific Rheumatoid Arthritis Risk Loci Reveal Limited Replication and Apparent Allelic Heterogeneity in North Indians
Source: PLoS One. 2012 Feb 15;7(2):e31584. doi: 10.1371/journal.pone.0031584 (PMC3280307; doi:10.1371/journal.pone.0031584)
Supplement: Table S2 — Test of association of additional (flanking) SNPs in European specific RA candidate genes/loci. (DOC) [file pone.0031584.s002.doc]

| **CHR** | **SNP** | **BP** | **A1** | **P** | **OR** | **L95** | **U95** |  | **Gene** |
| --- | --- | --- | --- | --- | --- | --- | --- | --- | --- |
| 1 | rs3789607 | 114366434 | G | 0.1402 | 1.101 | 0.9687 | 1.252 |  | PTPN22 |
| 1 | rs2476599 | 114363459 | A | 0.8439 | 0.9858 | 0.8547 | 1.137 |  | PTPN22 |
| 1 | rs1885800 | 117013732 | A | 0.1586 | 1.111 | 0.9598 | 1.285 |  | CD58 |
| 1 | rs4657039 | 161462728 | G | 0.6068 | 0.9674 | 0.8528 | 1.097 |  | FCGR2A |
| 1 | rs511278 | 161490896 | A | 0.06591 | 1.166 | 0.9899 | 1.374 |  | FCGR2A |
| 1 | rs3767742 | 198634997 | A | 0.09835 | 0.8742 | 0.7454 | 1.025 |  | PTPRC |
| 1 | rs7522020 | 198637853 | A | 0.1589 | 1.13 | 0.9533 | 1.339 |  | PTPRC |
| 1 | rs9803750 | 198644124 | A | **0.000438** | 0.79 | 0.6926 | 0.901 |  | PTPRC |
| 1 | rs16843670 | 198650006 | A | 0.275 | 0.8709 | 0.6794 | 1.116 |  | PTPRC |
| 1 | rs2359952 | 198656407 | G | **0.00016** | 0.7815 | 0.6875 | 0.8883 |  | PTPRC |
| 1 | rs3754098 | 198658856 | A | 0.09826 | 0.8774 | 0.7513 | 1.025 |  | PTPRC |
| 1 | rs12120762 | 198699793 | A | 0.9383 | 0.995 | 0.8768 | 1.129 |  | PTPRC |
| 1 | rs12127496 | 198723915 | C | 0.1062 | 0.8154 | 0.6363 | 1.045 |  | PTPRC |
| 1 | rs12409128 | 198733848 | A | 0.8879 | 1.018 | 0.791 | 1.311 |  | PTPRC |
| 1 | rs1932436 | 198739870 | A | 0.6683 | 0.9725 | 0.856 | 1.105 |  | PTPRC |
| 1 | rs7538393 | 198745866 | G | 0.722 | 0.9696 | 0.8179 | 1.149 |  | PTPRC |
| 1 | rs2784115 | 198751401 | A | 0.1506 | 0.9009 | 0.7814 | 1.039 |  | PTPRC |
| 1 | rs2784101 | 198757361 | G | 0.1705 | 1.11 | 0.9562 | 1.288 |  | PTPRC |
| 1 | rs2784103 | 198760554 | A | 0.0941 | 1.159 | 0.975 | 1.378 |  | PTPRC |
| 1 | rs1475169 | 198771204 | C | 0.9181 | 1.007 | 0.8854 | 1.145 |  | PTPRC |
| 1 | rs1575394 | 198785494 | G | 0.8142 | 0.9848 | 0.8665 | 1.119 |  | PTPRC |
| 1 | rs7520823 | 198798913 | G | 0.7306 | 1.028 | 0.8784 | 1.203 |  | PTPRC |
| 1 | rs12071005 | 198801107 | G | 0.2042 | 0.8676 | 0.6967 | 1.08 |  | PTPRC |
| 1 | rs10919593 | 198811372 | A | 0.09563 | 1.121 | 0.98 | 1.283 |  | PTPRC |
| 1 | rs12406238 | 198825960 | A | 0.3088 | 0.8688 | 0.6626 | 1.139 |  | PTPRC |
| 1 | rs10753838 | 198833320 | G | 0.2184 | 1.118 | 0.9362 | 1.334 |  | PTPRC |
| 1 | rs6701884 | 198837151 | G | 0.9266 | 0.9935 | 0.8643 | 1.142 |  | PTPRC |
| 1 | rs1322915 | 198864590 | G | 0.3871 | 0.9459 | 0.8338 | 1.073 |  | PTPRC |
| 1 | rs10489689 | 198889074 | G | 0.8147 | 0.9851 | 0.8689 | 1.117 |  | PTPRC |
| 1 | rs1040959 | 198895844 | C | 0.7769 | 0.9775 | 0.8351 | 1.144 |  | PTPRC |
| 1 | rs10919605 | 198898427 | G | 0.5606 | 1.04 | 0.9107 | 1.188 |  | PTPRC |
| 1 | rs11589679 | 198912797 | A | 0.6427 | 0.9517 | 0.7722 | 1.173 |  | PTPRC |
| 1 | rs2154329 | 198943840 | G | 0.8409 | 1.016 | 0.8673 | 1.191 |  | PTPRC |
| 1 | rs12408189 | 198959691 | G | 0.7306 | 1.028 | 0.8775 | 1.205 |  | PTPRC |
| 1 | rs10919614 | 198974825 | A | 0.8807 | 1.01 | 0.8891 | 1.147 |  | PTPRC |
| 1 | rs12240178 | 198976520 | G | 0.6427 | 0.9487 | 0.7594 | 1.185 |  | PTPRC |
| 1 | rs1036332 | 199012478 | A | 0.7044 | 0.9738 | 0.849 | 1.117 |  | PTPRC |
| 1 | rs10919618 | 199018143 | G | 0.4755 | 1.048 | 0.9211 | 1.193 |  | PTPRC |
| 1 | rs322931 | 199019855 | A | 0.7229 | 1.027 | 0.8869 | 1.189 |  | PTPRC |
| 2 | rs17533781 | 65522709 | A | 0.3776 | 1.087 | 0.9033 | 1.308 |  | SPRED2 |
| 2 | rs7561932 | 65545068 | G | 0.5135 | 1.043 | 0.9193 | 1.183 |  | SPRED2 |
| 2 | rs7601027 | 65545090 | G | 0.7317 | 1.025 | 0.8912 | 1.178 |  | SPRED2 |
| 2 | rs10170788 | 65546879 | A | 0.4997 | 1.044 | 0.9209 | 1.184 |  | SPRED2 |
| 2 | rs10185087 | 100424310 | G | 0.382 | 0.941 | 0.821 | 1.079 |  | AFF3 |
| 2 | rs7609179 | 100425448 | A | 0.9535 | 1.005 | 0.8388 | 1.205 |  | AFF3 |
| 2 | rs1429272 | 100427882 | C | 0.4821 | 0.9207 | 0.7311 | 1.159 |  | AFF3 |
| 2 | rs1366765 | 100441349 | G | 0.8558 | 0.9861 | 0.8485 | 1.146 |  | AFF3 |
| 2 | rs17023158 | 100447926 | G | **0.004862** | 1.453 | 1.119 | 1.886 |  | AFF3 |
| 2 | rs11677260 | 100455547 | A | 0.7963 | 0.9632 | 0.7244 | 1.281 |  | AFF3 |
| 2 | rs12473053 | 100457033 | G | 0.4896 | 1.062 | 0.8952 | 1.26 |  | AFF3 |
| 2 | rs13032879 | 100602759 | A | 0.8001 | 0.9798 | 0.8366 | 1.147 |  | AFF3 |
| 2 | rs11675333 | 100637331 | A | 0.122 | 0.8822 | 0.7526 | 1.034 |  | AFF3 |
| 2 | rs1115910 | 100647872 | A | 0.1031 | 1.11 | 0.979 | 1.259 |  | AFF3 |
| 2 | rs6706188 | 100739970 | G | **0.005** | 0.8125 | 0.703 | 0.939 |  | AFF3 |
| 2 | rs1437377 | 100744924 | C | **0.003** | 0.7776 | 0.6562 | 0.9214 |  | AFF3 |
| 2 | rs3181096 | 204570092 | G | 0.6225 | 1.033 | 0.9086 | 1.174 |  | CD28 |
| 2 | rs1181390 | 204572677 | A | 0.8203 | 1.024 | 0.8353 | 1.255 |  | CD28 |
| 2 | rs10490573 | 204583163 | A | 0.1807 | 1.186 | 0.9235 | 1.524 |  | CD28 |
| **2** | **rs4675367** |  | **A** | **0.041** | **1.8** | **1.02** | **3.19** |  | **CD28** |
| 2 | rs4673268 | 204639764 | A | 0.7797 | 1.035 | 0.8134 | 1.317 |  | CD28 |
| 2 | rs17187469 | 204648363 | G | 0.4928 | 1.088 | 0.8542 | 1.387 |  | CD28 |
| 2 | rs733618 | 204730944 | G | 0.8027 | 1.023 | 0.8569 | 1.221 |  | CTLA4 |
| 2 | rs231726 | 204740866 | A | **0.03486** | 1.158 | 1.01 | 1.328 |  | CTLA4 |
| 2 | rs231757 | 204753487 | C | 0.6612 | 0.9561 | 0.7823 | 1.169 |  | CTLA4 |
| 2 | rs10497873 | 204762327 | A | 0.3818 | 1.069 | 0.9202 | 1.243 |  | CTLA4 |
| 2 | rs10197319 | 204763044 | G | **0.03144** | 1.148 | 1.012 | 1.302 |  | CTLA4 |
| 3 | rs7622074 | 58312297 | G | **0.0109** | 1.178 | 1.038 | 1.336 |  | PXK |
| 3 | rs6445971 | 58314324 | C | 0.1395 | 0.8881 | 0.7587 | 1.04 |  | PXK |
| 3 | rs6445975 | 58370177 | C | 0.1688 | 0.9062 | 0.7876 | 1.043 |  | PXK |
| 3 | rs6787661 | 58378082 | A | 0.2189 | 0.8436 | 0.6432 | 1.107 |  | PXK |
| 3 | rs6767498 | 58409126 | A | **0.005488** | 0.8336 | 0.733 | 0.9479 |  | PXK |
| 4 | rs7685350 | 26229299 | A | 0.2827 | 1.091 | 0.931 | 1.277 |  | RBPJ |
| 4 | rs6831303 | 26275336 | A | 0.474 | 1.062 | 0.9004 | 1.253 |  | RBPJ |
| 4 | rs17644283 | 26308792 | G | 0.6479 | 1.03 | 0.9081 | 1.168 |  | RBPJ |
| 4 | rs946346 | 26431386 | A | 0.5892 | 1.053 | 0.8735 | 1.269 |  | RBPJ |
| 4 | rs3109848 | 26457168 | A | 0.2599 | 0.9135 | 0.7805 | 1.069 |  | IL2 |
| 4 | rs6848139 | 123395041 | C | **0.03017** | 1.331 | 1.027 | 1.724 |  | IL2 |
| 4 | rs4833834 | 123466351 | G | 0.4159 | 1.06 | 0.9206 | 1.222 |  | IL21 |
| 4 | rs6852535 | 123478716 | G | 0.5567 | 1.038 | 0.9157 | 1.178 |  | IL21 |
| 4 | rs1398553 | 123548068 | A | 0.4932 | 1.065 | 0.8899 | 1.274 |  | IL21 |
| 4 | rs17005934 | 123549699 | G | 0.7281 | 0.9667 | 0.7988 | 1.17 |  | IL21 |
| 4 | rs6840978 | 123554707 | A | 0.05 | 0.8319 | 0.6925 | 0.9993 |  | IL21 |
| 4 | rs2137497 | 123558254 | C | 0.4549 | 0.9491 | 0.8277 | 1.088 |  | IL21 |
| 4 | rs1512970 | 123561067 | A | 0.6679 | 0.9726 | 0.8568 | 1.104 |  | IL21 |
| 4 | rs1022234 | 123563078 | G | **0.01255** | 0.7975 | 0.6676 | 0.9528 |  | IL21 |
| 4 | rs10518405 | 123580354 | A | 0.4972 | 1.071 | 0.879 | 1.304 |  | IL21 |
| 4 | rs10518400 | 123582426 | A | 0.8176 | 1.024 | 0.8371 | 1.253 |  | IL21 |
| 5 | rs11742921 | 55349049 | A | 0.1477 | 1.108 | 0.9643 | 1.273 |  | ANKRD55 |
| 5 | rs6877664 | 55401335 | A | **0.01649** | 1.413 | 1.064 | 1.876 |  | ANKRD55 |
| 5 | rs160919 | 55409421 | A | 0.1222 | 1.111 | 0.9722 | 1.269 |  | ANKRD55 |
| 5 | rs10214316 | 55444040 | G | **0.004891** | 1.204 | 1.058 | 1.37 |  | ANKRD55 |
| 5 | rs7706204 | 55477779 | G | 0.2156 | 1.137 | 0.9279 | 1.393 |  | ANKRD55 |
| 5 | rs159574 | 55479662 | G | 0.4053 | 1.055 | 0.9297 | 1.198 |  | ANKRD55 |
| 5 | rs36747 | 55488928 | C | 0.2132 | 1.088 | 0.9529 | 1.241 |  | ANKRD55 |
| 5 | rs149140 | 55516988 | A | **0.007891** | 1.186 | 1.046 | 1.346 |  | ANKRD55 |
| 5 | rs32498 | 55548797 | G | **0.005913** | 1.196 | 1.053 | 1.359 |  | ANKRD55 |
| 5 | rs11242493 | 102714963 | C | 0.4231 | 0.8979 | 0.6897 | 1.169 |  | C5orf30 |
| 5 | rs6596533 | 102739759 | A | 0.7663 | 1.027 | 0.8619 | 1.224 |  | C5orf30 |
| 6 | rs654039 | 138115266 | G | 0.4342 | 0.9503 | 0.8365 | 1.08 |  | TNFA1P3 |
| 6 | rs3757173 | 138190154 | G | **0.03714** | 1.261 | 1.014 | 1.569 |  | TNFA1P3 |
| 6 | rs5029936 | 138194937 | A | **0.03015** | 1.338 | 1.028 | 1.743 |  | TNFA1P3 |
| 6 | rs9389541 | 138253479 | C | 0.7707 | 0.974 | 0.8158 | 1.163 |  | TNFA1P3 |
| 6 | rs12216269 | 138262537 | C | 0.1279 | 1.106 | 0.9715 | 1.259 |  | TNFA1P3 |
| 6 | rs3117148 | 159453813 | G | 0.1218 | 0.8715 | 0.7321 | 1.037 |  | TAGAP |
| 6 | rs926657 | 159463452 | A | **0.002972** | 0.6526 | 0.4915 | 0.8664 |  | TAGAP |
| 6 | rs9295089 | 159463964 | G | **0.008926** | 0.7081 | 0.5461 | 0.9182 |  | TAGAP |
| 6 | rs1738074 | 159465977 | A | 0.1256 | 0.9041 | 0.7946 | 1.029 |  | TAGAP |
| 6 | rs380885 | 159467351 | A | 0.2349 | 0.9048 | 0.767 | 1.067 |  | TAGAP |
| 6 | rs212402 | 159472295 | G | **0.008739** | 0.8158 | 0.7006 | 0.95 |  | TAGAP |
| 6 | rs2073137 | 159475468 | A | 0.08961 | 1.165 | 0.9766 | 1.389 |  | TAGAP |
| 6 | rs1331301 | 167502638 | C | **0.01344** | 0.8487 | 0.7452 | 0.9666 |  | CCR6 |
| 6 | rs150110 | 167511766 | G | 0.6338 | 0.9673 | 0.8438 | 1.109 |  | CCR6 |
| 6 | rs4710181 | 167513998 | G | 0.2154 | 1.083 | 0.9544 | 1.23 |  | CCR6 |
| 6 | rs1556413 | 167524743 | A | **0.02075** | 0.8607 | 0.758 | 0.9774 |  | CCR6 |
| 8 | rs4841548 | 11374779 | G | **0.03011** | 0.826 | 0.6949 | 0.9819 |  | BLK |
| 8 | rs2618451 | 11376266 | A | 0.133 | 0.9054 | 0.7952 | 1.031 |  | BLK |
| 8 | rs2618450 | 11376790 | A | 0.9238 | 1.007 | 0.8672 | 1.17 |  | BLK |
| 8 | rs12677843 | 11387189 | A | 0.458 | 1.05 | 0.9225 | 1.196 |  | BLK |
| 8 | rs17806523 | 11406175 | A | **0.009095** | 0.7303 | 0.5763 | 0.9256 |  | BLK |
| 8 | rs2256647 | 11491263 | G | 0.2132 | 0.9211 | 0.8092 | 1.048 |  | BLK |
| 9 | rs7024727 | 34730395 | A | 0.9233 | 1.008 | 0.8524 | 1.193 |  | CCL21 |
| 9 | rs10758270 | 34730754 | G | 0.6984 | 0.9717 | 0.8406 | 1.123 |  | CCL21 |
| 10 | rs12722589 | 6060423 | C | **7.41E-05** | 1.481 | 1.217 | 1.802 |  | IL2RA |
| 10 | rs12722588 | 6060433 | A | **0.008263** | 0.792 | 0.6659 | 0.9418 |  | IL2RA |
| 10 | rs11256442 | 6079344 | A | 0.103 | 1.113 | 0.9786 | 1.265 |  | IL2RA |
| 10 | rs11256448 | 6079479 | G | 0.9491 | 1.005 | 0.872 | 1.157 |  | IL2RA |
| 10 | rs706779 | 6098824 | G | 0.2072 | 0.9195 | 0.807 | 1.048 |  | IL2RA |
| 10 | rs3134883 | 6100725 | A | 0.3989 | 1.059 | 0.9269 | 1.21 |  | IL2RA |
| 10 | rs3118470 | 6101713 | G | **0.002051** | 1.219 | 1.075 | 1.383 |  | IL2RA |
| 10 | rs947473 | 6389914 | A | 0.4477 | 0.9374 | 0.7932 | 1.108 |  | PRKCQ |
| 20 | rs6032661 | 44733811 | G | 0.6365 | 1.038 | 0.8889 | 1.212 |  | CD40 |
| 20 | rs6065925 | 44734296 | A | **0.004783** | 1.212 | 1.06 | 1.385 |  | CD40 |
| 20 | rs1535044 | 44739156 | A | 0.1093 | 0.8823 | 0.7569 | 1.028 |  | CD40 |
| 20 | rs3746821 | 44755111 | A | 0.4922 | 0.9403 | 0.7888 | 1.121 |  | CD40 |
| 20 | rs11569333 | 44755889 | A | 0.2318 | 0.8639 | 0.6796 | 1.098 |  | CD40 |
| 22 | rs228942 | 37524619 | A | **0.04425** | 0.8363 | 0.7025 | 0.9956 |  | IL2RB |
| 22 | rs84460 | 37525731 | G | 0.1023 | 0.8859 | 0.766 | 1.025 |  | IL2RB |
| 22 | rs228947 | 37526529 | A | 0.1592 | 0.8739 | 0.7243 | 1.054 |  | IL2RB |
| 22 | rs3218322 | 37528098 | A | 0.5424 | 1.041 | 0.9154 | 1.183 |  | IL2RB |
| 22 | rs3218315 | 37529724 | A | 0.4962 | 1.046 | 0.9187 | 1.191 |  | IL2RB |
